# Acquisition of the capsule locus by horizontal gene transfer in *Neisseria meningitidis* is often accompanied by the loss of UDP-GalNAc synthesis

**DOI:** 10.1038/srep44442

**Published:** 2017-03-14

**Authors:** Stephanie N. Bartley, Shakeel Mowlaboccus, Christopher A. Mullally, Keith A. Stubbs, Alice Vrielink, Martin C. J. Maiden, Odile B. Harrison, Timothy T. Perkins, Charlene M. Kahler

**Affiliations:** 1 School of Biomedical Sciences, University of Western Australia, Perth, Australia; 2 The Marshall Centre for Infectious Disease Research and Training, University of Western Australia, Perth, Australia; 3 School of Molecular Sciences, University of Western Australia, Perth, Australia; 4 University of Oxford, Department of Zoology, South Parks Road, Oxford OX1 3PS, United Kingdom

## Abstract

Pathogenic meningococci have acquired a 24 kb capsule synthesis island (*cps*) by horizontal gene transfer which consists of a synthetic locus and associated capsule transport genes flanked by repetitive Regions D and D’. Regions D and D’ contain an intact gene encoding a UDP-galactose epimerase (*galE1*) and a truncated remnant (*galE2*), respectively. In this study, GalE protein alleles were shown to be either mono-functional, synthesising UDP-galactose (UDP-Gal), or bi-functional, synthesising UDP-Gal and UDP-galactosamine (UDP-GalNAc). Meningococci possessing a capsule null locus (*cnl*) typically possessed a single bi-functional *galE*. Separation of functionality between *galE1* and *galE2* alleles in meningococcal isolates was retained for all serogroups except serogroup E which has a synthetic requirement for UDP-GalNAc. The truncated *galE2* remnant in Region D’ was also phylogenetically related to the bi-functional *galE* of the *cnl* locus suggesting common ancestry. A model is proposed in which the illegitimate recombination of the *cps* island into the *galE* allele of the *cnl* locus results in the formation of Region D’ containing the truncated *galE2* locus and the capture of the *cps* island en bloc. The retention of the duplicated Regions D and D’ enables inversion of the synthetic locus within the *cps* island during bacterial growth.

The *Neisseria* genus contains eleven species that colonize the oropharyngeal and urogenital mucosa of humans and of these, *Neisseria gonorrhoeae* and *N. meningitidis*, are of medical importance. *N. gonorrhoeae* colonises the mucosal surfaces of the urogenital tract in both males and females^1^. *N. meningitidis* is predominantly an opportunistic pathogen, asymptomatically colonising the mucosa of the nasopharynx of approximately 10% of the adult population^2^,^3^ but occasionally invades the host, resulting in septicaemia and meningitis^4^.

In both *N. meningitidis* and *N. gonorrhoeae,* interactions with host cells are modulated by the glycome on the bacterial surface which includes the lipooligosaccharide (LOS) and the glycosylation status of the type IV pilin^5^. Meningococci are distinguished from gonococci by the presence of a capsule polysaccharide synthesis (*cps*) island which encodes a capsule that facilitates systemic infection^5^. The *cps* island has a lower G/C-content than the core meningococcal genome consistent with acquisition via horizontal gene transfer (HGT) by recombination^6^. The general organisation of the *cps* island is as follows: Region A is responsible for the synthesis of the capsule polymer; Region B (*ctrEF*) and Region C (*ctrABCD*) contain genes responsible for transporting the capsule polymer to the bacterial surface while Region D and Region D’ contain an intact and truncated remnant copy of a gene encoding UDP-galactose epimerase, known as *galE1* and *galE2*, respectively. Region E contains a gene of unknown function termed *tex* which is a homologue of a transcription factor. GalE is necessary for the synthesis of UDP-galactose (UDP-Gal) which is utilised in for the synthesis of capsule polymers from serogroup W/Y, LOS and protein glycosylation^5^.

Non-pathogenic meningococci frequently possess a capsule null locus (*cnl*) with a single copy of Region D with an intact *galE* and Region E (Fig. 1)^7^. Evolutionary studies have suggested that meningococci acquired the *cps* island from potential donors such as *Pasteurella multocida* and *Haemophilus influenzae* through mosaic HGT events^8^,^9^,^10^. Although the ancestral events leading to the formation of the *cps* island are unclear, serogroup switching can result in the replacement of the entire synthetic Region A in instances where serogroups B or C switch to serogroups A, W or Y (Fig. 1) indicating recombination events in this region continue to occur^11^,^12^.

Gonococci also possess a *cnl* locus (Fig. 1), and have the additional ability to synthesise UDP-*N*-acetyl galactosamine (UDP-GalNAc) which is used to terminate the non-reducing terminus of LOS^13^. In meningococci, GalNAc is not a component of LOS^5^,^14^, but is required for the synthesis of meningococcal serogroup E and Z capsules^15^,^16^. Although many of the biosynthetic pathways of *Neisseria* spp. have been elucidated, the mechanism by which UDP-GalNAc is synthesised has not been proposed. Bacteria synthesize UDP-GalNAc via either a bifunctional UDP-galactose 4-epimerase (GalE) or a UDP-GalNAc 4-epimerase (GNE). Since *Neisseria* spp. do not harbour a GNE homologue, the neisserial GalE1 could be a bi-functional epimerase that synthesizes both UDP-Gal and UDP-GalNAc from the substrates UDP-glucose (UDP-Glc) and UDP- *N*-acetylglucosamine (UDP-GlcNAc), respectively.

This study examined the functional diversity of the *galE* locus and found that *Neisseria* spp. possess both mono-functional and bi-functional UDP-galactose epimerases. While *N. gonorrhoeae* and meningococci with a *cnl* locus possess bi-functional *galE* alleles which are phylogenetically related, meningococci carrying the *cps* locus possessed both bi- and mono-functional alleles for *galE1* and *galE2*. The phylogenetic relations of the *galE* alleles and their associative properties with serogroup and clonal complex has provided further evidence for the hypothetical model of the recombination events during HGT of the *cps* island.

## Results

### *galE1* alleles form two distinct phylogenetic clusters in *Neisseria* species

The distribution of *galE1* alleles (NEIS0048) across the genus *Neisseria* was assessed using a defined set of 194 isolates for which genomic data was available in the PubMLST database (www.pubmlst.org/neisseria, Supplementary Table S1). A total of 107 unique *galE* alleles, comprising 65 *galE1* alleles from meningococci and 42 *galE* alleles from the other species, represented the top 24 most commonly occurring NEIS0048 alleles within the PubMLST database ( > 0.38% occurrences). An un-rooted neighbour-net tree (Fig. 2) revealed that the alleles formed two main clusters. Cluster A contained encapsulated meningococci (*p-*distance = 0.092), while cluster B contained *N. gonorrhoeae* and the non-pathogenic *Neisseria* species, *N. lactamica* and *N. polysaccharea* as well as unencapsulated meningococci possessing the *cnl* locus (overall mean *p-*distance = 0.063) (Fig. 2). Several other less distinct clusters with deeper roots were observed which corresponded with *N. subflava, N. oralis, N. elongata,* and *N. cinerea*. Two meningococcal *galE1* variants belonging to serogroup E and Z meningococci were found on the same branch as those from *N. animalis* and *N. weaveri* which were more distantly related to Cluster A and B (overall *p-*distance = 0.266). Overall, the distribution of the *galE1* alleles into at least two distinct clusters suggest that these alleles are under diversifying selection which may be related to function between species.

### *Neisseria* spp. possess mono- and bi-functional GalE epimerases

To investigate whether NEIS0048 alleles from each phylogenetic cluster had different functions, the *galE1* of *N. gonorrhoeae* strain FA1090 (GalE1 allele 17 = GalE_17) from cluster B and *N. meningitidis* strain MC58 (GalE1 allele 2 = GalE_2) from cluster A were cloned into pET15b to create GalE_17::Hisx6 and GalE_2::Hisx6. GalE_17::Hisx6 could epimerise both UDP-Glc and UDP-GlcNAc indicating that this variant is bi-functional, with the equilibrium favouring the production of UDP-Gal and UDP-GalNAc (Table 1). In comparison, GalE_2::Hisx6 would only accept UDP-Glc as a substrate and could not epimerise UDP-GlcNAc indicating that this enzyme is mono-functional. Unlike the gonococcal GalE_17 allele, the meningococcal GalE_2 had an equilibrium favouring the formation of UDP-Glc at 63% of all products.

### Mono- and bi-functionality of GalE epimerases is determined by a single amino acid residue in the active site

Previous investigations of known mono-functional and bi-functional GalE proteins have shown that a single amino acid residue is the main factor in the ability of the enzyme to possess singular or dual modes of activity. Thoden *et al*.^17^ compared the GalE protein from *H. sapiens* (Group 2) to the *E. coli* (Group 1) homologue and demonstrated that four conserved residues hold the sugar in a productive conformation (Supplementary Fig. 1) while a fifth residue affected whether UDP-Glc or UDP-GlcNAc could enter the active site cleft. In *E. coli* GalE and the human GalE, this position is occupied by a tyrosine (Y299) residue or a cysteine (C307), respectively. When the tyrosine residue of *E. coli* GalE was mutated to cysteine (Y299C) it switched the native enzyme from a mono-functional to a bi-functional mode while the converse occurred in *Yersinia enterocolitica* GalE^18^.

To understand the contribution of amino acid residues to the active site of GalE, 107 *galE* alleles from ten *Neisseria* spp. (Supplementary Table S1) were translated and aligned using CLUSTALW2^19^. As expected, residues K85, S125, Y150 and N180 which correspond to the four residues co-ordinating the pyranose ring in the active site were absolutely conserved (Supplementary Fig. 1). The fifth position was occupied by a serine (S299) residue in gonococcal GalE amino acid sequences and phenylalanine (F300) in most meningococcal alleles. Since the serine and phenylalanine residues are similar in size to cysteine in bi-functional enzymes and tyrosine in mono-functional enzymes, respectively, we proposed that these residues govern the functionality of GalE. Using PHYRE2^20^, homology models using GalE amino acid sequences of *N. gonorrhoeae* strain FA1090 and *N. meningitidis* strain MC58 were built. These models and the crystal structures of the *E. coli* and human enzymes in the presence of NADH and UDP-GlcNAc are shown in Fig. 3. To address whether variability within the amino acid sequences of neisseria GalE proteins contributed to the binding site pocket, 38 non-conserved residues derived from the alignment of 103 GalE1 alleles (excluding the four most distantly related alleles from *N. weaveri* GalE_115, *N. animalis* GalE_110, and *N. meningitidis* GalE_14 and GalE_15) were mapped to the predicted structure of GalE_2 from *N. meningitidis* MC58 (Fig. 3A). The non-conserved residues were localized predominantly to the external surface of the protein and were distant from the active site. Conservation, both in structure and in ligand binding residues, was evident for all four enzymes with the exception of positions F300 in meningococci and S299 in gonococci. Comparison of the structures suggests that when a small residue is present (C307 in humans or S299 in gonococci), both the UDP-GlcNAc and UDP-Glc are able to bind productively and hence the enzyme is bi-functional (Fig. 3C,E). In contrast, in the presence of an aromatic side chain (Y299 in *E. coli* or F300 in meningococci), the UDP-GlcNAc does not bind correctly in the active site to allow efficient hydride transfer from the sugar moiety to the nicotinaimide and, thus the enzyme is mono-functional for only UDP-Glc (Fig. 3B,D).

To test the role of S299 and F300 in determining substrate specificity of neisserial GalE enzymes, GalE_17(S299F)::Hisx6 and GalE_2(F300S)::Hisx6 mutants were constructed using site-directed mutagenesis and these recombinant enzymes were purified. The GalE_17(S299F)::Hisx6 lost the ability to epimerise UDP-GlcNAc while retaining the ability to convert UDP-Glc to UDP-Gal, although the equilibrium was altered to favour the formation of UDP-Glc compared to the wild-type enzyme (from 39% to 59%). Meningococcal GalE_2(F300S)::Hisx6 gained the ability to epimerise both UDP-Glc and UDP-GlcNAc although the observed equilibrium resulted in less UDP-GalNAc for GalE_2(F300S)::Hisx6 than gonococcal GalE_17::Hisx6 (Table 1).

### The distribution of mono- and bi- functional GalE1 (NEIS0048) is related to species

A Neighbor-Joining phylogenetic tree of 89 GalE1 amino acid sequences (Supplementary Table S1, Supplementary Fig. 2) revealed at least three major lineages Clades 1, 2, and 3, in addition to two sets of deeply branched outliers. One group of outliers contained alleles from *N. weaveri, N. animalis* with serogroup E and serogroup Z expressing meningococci. The GalE1 alleles from the serogroup E and serogroup Z expressing meningococci both have a cysteine in the active site cleft consistent with the proposed bi-functionality of GalE1 and the requirement for UDP-GalNAc for capsule synthesis. The GalE from *N. weaveri* also contained a cysteine in the active site cleft and was predicted to be bi-functional. The GalE proteins from *N. animalis* and *N. subflava* were both predicted to be mono-functional due to the presence of a tyrosine or phenylalanine residue, respectively, in the active site cleft.

Clade 1 containing GalE amino acid alleles from *N. elongata* and *N. mucosa,* was predicted to be bi-functional due to the presence of either a valine or a cysteine residue in the active site cleft. Clade 2 contained alleles from *N. cinerea, N. polysaccharea, N. lactamica, N. gonorrhoeae, N. bergeri*, and five meningococci. All of these were assigned bi-functionality due to an active site serine residue characteristic of the bi-functional alleles of *N. gonorrhoeae*. GalE alleles 16, 24, 37, 129 and 166 were present in meningococcal *cnl* isolates. Clade 3 contained only GalE1 amino acid sequences from *N. meningitidis* which were either mono- (contained a phenylalanine in the active site) or bi-functional (contained a serine or cysteine in the active site). Two distantly related protein alleles from *N. meningitidis,* GalE_41 and GalE_34, from a serogroup A and a serogroup C isolate, respectively, were most similar to protein alleles from *N. subflava*. The bi-functional alleles in clade 3 were associated with cc174 isolates expressing serogroup Y capsules (allele 131), a variety of serogroup B isolates from various genetic lineages (alleles 46, 49 and 236), a cc1 isolate expressing serogroup A capsule (allele 50), and serogroup I and K expressing isolates (allele 4 and 5 respectively). GalE_236 was selected as a representative of the bi-functional alleles in clade 3 for assessment of epimerase functionality. It was cloned, expressed as a His-tagged protein, assessed by HPLC and was shown to be bi-functional (Table 1).

### Region D and Region D’ of encapsulated meningococci generally encode mono- and bi-functional GalE, respectively

**Figure 1:**
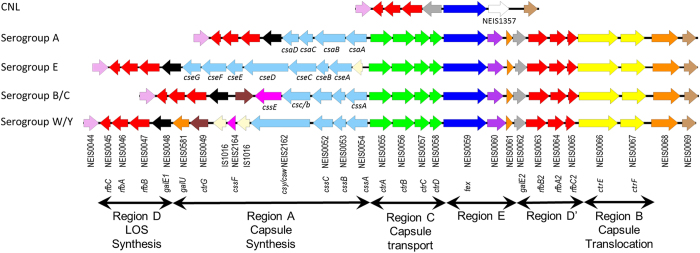
Genetic organisation of *cnl* and *cps* loci in *N. meningitidis*. Schematic organisation of the capsule synthesis (*cps*) island in meningococci. Serogroups A, B/C, W/Y and E are represented. Region A (light blue) is accompanied by Region C (bright green) encoding four capsule transport encoding genes *ctrA-D*. Region E (navy) encodes *tex*, a gene of unknown function homologue of a transcription factor. Region A-C-E is flanked by an inverted repeat segment, Region D and Region D’. Region D contains *galE1* (black), encoding the UDP-galactose 4-epimerase and three genes termed *rfbBAC* (red) of unknown function. Region D’ contains a truncated *galE2* (grey) and *rfbB2-A2-C2* (red). Region B (yellow) contains *ctrE* and *ctrF* which encode KDO transferases to initiate polysaccharide biosynthesis. Region A (light blue) contains polysaccharide biosynthesis genes specific for each serogroup and *ctrG* (brown) which is found in *cps* islands encoding sialic acid biosynthesis pathways. The four genes *csaA, csaB, csaC* and *csaD* are required for the synthesis of a homopolymer of *O*-acetylated, α1 → 6-linked ManNAc 1-phosphate of serogroup A meningococci. Serogroup B, C, W and Y meningococci contain three genes required for sialic acid biosynthesis, *cssA, cssB* and *cssC*. Each cluster has a serogroup specific polysialyltransferase, capsule synthesis serogroup B (*csb*), capsule synthesis serogroup C (*csc*), capsule synthesis serogroup W (*csw*) and capsule synthesis serogroup Y (*csy*). Serogroup C polysaccharides are O-acetylated by CssE while serogroup Y/W are acetylated by CssF. The biosynthesis cluster for serogroup W and Y also contains a *galU* encoding a UTP:α-D-glucose-1-phosphate uridylyltransferase. Serogroup B consists of a homolpolymer of α2 → 8 linked sialic acid, serogroup C consists of α2 → 9 linked sialic acid, serogroup W consists of repeating units of 4-*O*-α-D-galactopyranosyl-β-D-*N*-acetylneuraminic acid and serogroup Y consists of repeating units of 4-*O*-α-D-glucopyranosyl-β-D-*N*-acetylneuraminic acid. Serogroup E polysaccharide consists of alternating D-galactosamine and 2-keto-3-deoxyoctulosonate (KDO) residues synthesized by the seven genes *cseA-G*. The capsule null locus (*cnl*) of non-disease causing meningococci consists of *galE-rfbBAC* and *tex*. The figure was drawn using Easyfig^50^ and is similar to the figure presented by Harrison *et al*.^46^.

In addition to Region D containing the *galE1* allele, encapsulated meningococci also possess the inverted repeat Region D’ which contains a truncated remnant *galE2* in an operon with *rfbBAC’* (Fig. 1). The *galE2* pseudogene has lost the first 402 nucleotides but retains the last 615 nucleotides, containing the active site motif which can be used to assign ancestral functionality. To unambiguously assign a function to *galE2* alleles, *galE1* and *galE2* genes were manually curated in 1196 meningococci representing 10 common clonal complexes. This yielded 65 *galE1* alleles and 24 *galE2* alleles (data not shown). Of the 65 *galE1* alleles in 1196 isolates, 75% were associated with mono-functionality (F300) with the remainder being bi-functional (S300). The GalE1 alleles from serogroups B, W and Y were predominantly mono-functional (Fisher’s exact test, two-tailed, *p* < 0.05). However, no significant association of GalE1 functionality was noted for serogroups A and C although the trend in this small dataset was towards mono-functionality (Supplementary Table S2). Bi-functional GalE1 alleles were associated with all serogroup E expressing isolates (Fisher’s exact test, two-tailed, *p* < 0.0001) due to the requirement for UDP-GalNAc for the synthesis of this capsule polymer. Of the 24 *galE2* alleles, 71% were associated with bi-functionality with the remainder being mono-functional. Overall, 92.4% of meningococcal strains carried a mono-functional *galE1* and a bi-functional remnant *galE2* allele. There is a strong retention (Fisher’s exact test, two-tailed, *p* < 0.05 for each serogroup) of separate ancestral functionality between *galE1* and *galE2* alleles in meningococcal isolates that expressed capsules other than serogroup E.

### Recombination rates are highest in *galE1, galE2* and *tex* in the *cps* island

Since meningococcal *galE1* and *galE2* are highly conserved at the nucleotide level, homologous recombination between the two genes may randomly switch functionality between *galE1* and *galE2* loci. To examine this further, recombination events per mutation (r/m) was used to calculate the number of recombination events relative to mutations (*ρ/θ*) and the recombination events relative to the number of mutations within *galE1* and *galE2* with respect to other *cps* island regions (Supplementary Table S3, Fig. 4). Recombination events occurred twice as often as point mutations in *galE1*, 1.5 times as often in *ctrA-D* and *tex* while recombination and mutation equally contributed to diversity within *ctrG, galE2* and *ctrEF.* Diversity within NEIS0044, located on the boundary of Region D containing *galE1*, was equally driven by recombination and mutation, whereas diversity of NEIS0069 on the boundary of Region B was mostly caused by mutation (Fig. 4A). When recombination rates were corrected for local block boundaries, *galE1, tex* and *galE2* had the highest recombination rates relative to the other genes in the *cps* locus (Fig. 4B). Therefore, although recombination does contribute to the diversity of *galE1* and *galE2* alleles, the exchange of functionality between the two loci is infrequent since the functional separation of the two loci is maintained in the clonal complexes that do not possess serogroup E.

### Meningococcal *galE2* represents a potential site for the capture of the *cps* island

The origin of Region D’ containing *galE2* is currently unknown. While the majority of encapsulated meningococci contained *galE2* with a motif consistent with an ancestral bi-functional activity, most *cnl* meningococci from various genetic lineages including cc53 and cc198 possessed bi-functional alleles (GalE_37, GalE_16 and GalE_116, GalE_161, GalE_281, GalE_362 alleles, GalE_378, and GalE_397). An alignment of the conserved portions of the *galE1* and *galE2* alleles revealed a bi-furcated phylogeny with the bi-functional *galE1/galE2* alleles and mono-functional alleles forming separate clusters (Fig. 5). Interestingly, the *galE* alleles from *cnl* meningococci clustered with the bi-functional *galE2* alleles. This observation generated the hypothesis that the *galE2* of Region D’ in pathogenic meningococci is a remnant of *galE* from the original *cnl* locus of the ancestral recipient genome during the capture of the *cps* island. The *cnl* site is always located between two boundaries: NEIS0044 upstream of the *galE1*-*rfbBAC* cluster and NEIS0069 downstream of *tex* (Fig. 1). The capture of the *cps* island was postulated to lead to the deletion of the 5′ end of *galE-tex-* NEIS1357 in the *cnl* locus to create NEIS0044-*rfbCAB*’-*galE2* linked to the *cps* island ending at the downstream boundary of NEIS0069. In this model, the *cps* island HGT event would require the introduction of Region A-C-E-D-B by recombination into the *galE* of the *cnl* locus.

One outcome of this model would be the expectation of strong linkage disequilibrium between the two *galE* loci with a strong association with serogroup as these would be linked en bloc during the HGT event. A plot of the association of *galE1* and *galE2* allelic pairs with serogroup in 1196 isolates representing each of the 10 clonal complexes showed that each clonal complex was characterized by a predominant *galE1*/serogroup/*galE2* combination (Supplementary Fig. S3). In summary, 84.2% of cc1 is represented by GalE1_39_/GalE2_30_, 74.2% of cc11 is represented by GalE1_10_/GalE2_8_, 46.6% of cc22 is represented by GalE1_8_/GalE2_13_, 88.8% of cc23 is represented by GalE1_27_/GalE2_23_, 73.2% of cc41/44 is represented by GalE1_20_/GalE2_13_, 65.3% of cc60 is represented by GalE1_10_/GalE2_13_, and 78.4% of cc269 is represented by GalE1_120_/GalE2_7_. A strong associative property with clonal complex was observed for *galE1/galE2* allelic pairs (Cramer’s V coefficient, V = 0.985), *tex* (V = 0.963), *ctrE* (V = 0.948) and *ctrF* (V = 0.770) suggesting that the correlation of alleles in the *cps* island with a specific clonal complex was formed independently during the acquisition of the *cps* island into the founder of each clonal complex.

This hypothetical model for the acquisition of the *cps* island in modern clonal complexes also predicted that the original organization of the *cps* island upon acquisition could have been NEIS0044-Region D’ (containing *galE2* bi-functional)-E-C-A-D (*galE1* mono-functional)-B. However, the most common arrangement found in closed genomes of strains MC58 and FAM18 has NEIS0044 followed by Region D containing *galE-* A- C- E- D’- B- NEIS0069 (Fig. 6) which suggests that the synthesis cassette, A-C-E, may undergo inversion in this site. Colony PCR confirmed that both *cps* island arrangements were detectable in plate grown cultures of strains MC58, FAM18 and NMB (Supplementary Fig. S4). Complete sequencing of these PCR products did not detect recombination events within the *galE1* and *galE2* genes which is consistent with potential breakpoints in the flanking *rfbBAC* gene clusters of Region D and Region D’. However, since the paired *rfbBAC* and *rfbBAC’* regions in each strain was almost identical, the recombination site was not detected.

### Meningococcal *galE1* and *tex* represent potential sites for homologous recombination during serogroup switching events

Mustapha *et al*.^12^ have recently described the recombination events detected upon the integration of the serogroup W cassette into the cc11 lineage which originally expressed serogroup C (Fig. 1). They showed that the left hand boundary for the integration of Region A for serogroup W synthesis occurred in *galE1* while *galE2* remained unchanged. To examine whether this is a common occurrence in serogroup switching, the association of the *galE1/galE2* allelic pairs with serogroup was plotted (Supplementary Fig. S3). The dominating serogroup in each lineage is strongly associated with only one *galE1/galE2* allelic pair. For example, 93.5% of cc11:serogroup C isolates possessed GalE1_27/GalE2_8 allelic pair whereas 100% of cc11:serogroup W isolates possessed GalE1_10/GalE2_8. Capsule switching events which involved the acquisition of serogroup B into cc11 displayed a similar pattern with 75% retaining GalE2_8 while the allele of GalE1 with Region A had been changed. Similarly, 16 events in cc60:serogroup E strains involving the acquisition of serogroup B synthetic regions showed a similar pattern of variation in GalE1 with conservation of the GalE2 alleles (Supplementary Table S4). Although the library of 1196 isolates contained 65 unique GalE1/GalE2 allelic pairs, only four GalE1/GalE2 pairs were found in multiple clonal complexes (Supplementary Fig. 3) suggesting that these exchanges had recombination boundaries outside of the *galE1/galE2* pairs.

## Discussion

The acquisition of the *cps* island by HGT into an ancestral non-pathogenic meningococcal lineage carrying a *cnl* locus similar to cc53 and cc198 isolates of *N. meningitidis* has been proposed by Schoen *et al*.^6^ and others^21^,^22^,^23^,^24^,^25^. This hypothetical model proposes that the modern arrangement of the *cps* island is the outcome of at least two recombination events that resulted in the capture of Region A-C and Region B, located upstream and downstream of Region E (*tex*), respectively, in the capsule null locus of non-pathogenic meningococci^8^,^9^,^10^,^26^. In this study, we provide evidence for a second hypothetical model which suggests, that once the ancestral *cps* island was formed, this structure became the donor sequence for en bloc transfer into the modern clonal complexes of *N. meningitidis*. Evidence for this hypothetical model is based upon the observation that the *galE1* and *galE2* alleles are phylogenetically distinct due to different enzymatic functions, and that these phylogenetic relationships can be traced through capsule null loci and in *cps* islands in the various clonal complexes of *N. meningitidis*.

Gonococci have a requirement for UDP-GalNAc which is a component of the gonococcal LOS^27^. The gonococcal GalE epimerase was shown to be bi-functional with the capacity to synthesise both UDP-Gal and UDP-GalNAc whereas the meningococcal GalE could only produce UDP-Gal. Exchanging a pivotal amino acid in the active site cleft (F300 in meningococci and S299 in gonococci) enabled the exchange of mono- and bi-functionality of the enzymes suggesting that the identity of the amino acid positioned at the mouth of the active site cleft determined the functional phenotype of the epimerase. Ishiyama *et al*.^28^ proposed that changing the amino acid at this position of the active site cleft of GalE epimerase determined the ability of the UDP-linked *N*-acetyl-sugar to rotate and undergo epimerisation. Although there are other non-conserved amino acid positions within neisserial GalE variants, none of these were shown to influence the cleft leading to the active site (Fig. 3). Therefore, the use of the six amino acid residue motif surrounding the active site cleft residue of F300 (meningococci)/S299 (gonococci) or equivalent in commensal *Neisseria* spp. was considered sufficient to assign functionality to the *galE* alleles.

**Figure 2:**
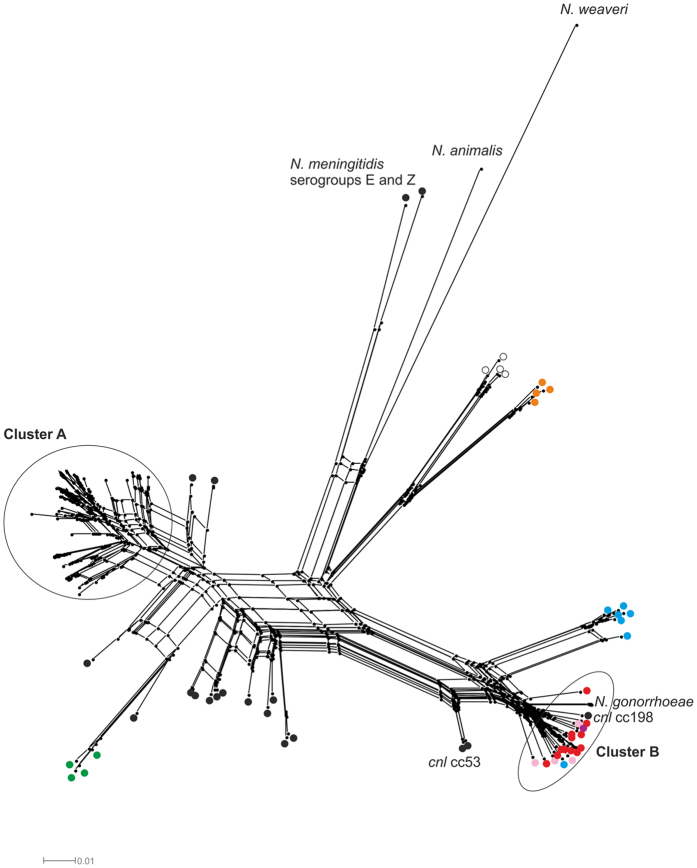
Phylogenetic reconstruction using *galE* alleles from commensal and pathogenic *Neisseria* spp. using an un-rooted neighbour-net algorithum. *N. gonorrhoeae* and commensal species are represented by circles with different colours denoting species. These were: *N. meningitidis* is black; *N. lactamica* is red: *N. polysacchareae* is pink; *N. bergeri* is mauve; *N. cinerea* is blue; *N. elongata* is orange*; N. animalis* and *N. weaveri* are grey; *N. mucosa* is white and *N. subflava* is green. *N. meningitidis* isolates are found in cluster A but also on branches in-between clusters A and B as well as the *cnl* in cluster B. *N. gonorrhoeae* are only found in one branch of cluster B and are labelled as *N. gonorrhoeae* without a symbol.

**Figure 3:**
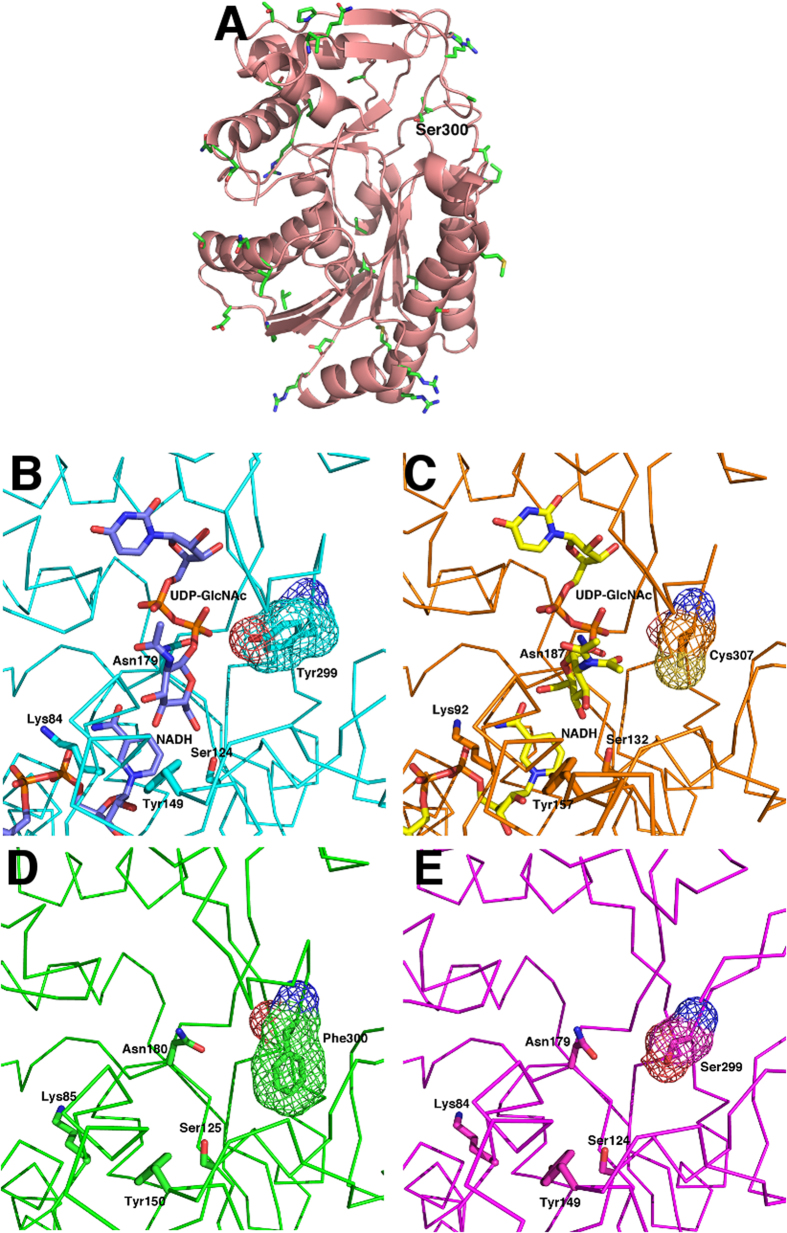
Structural views of mono-functional and bi-functional GalE from different organisms. (**A**) The predicted structure of GalE from *N. meningitidis* MC58 obtained using PHYRE2. The structure is depicted as a ribbon representation showing the secondary structure elements. The side chains for residues which are not conserved amongst all neisseria species are shown in stick representation. Position 300 in *N. gonorrhoeae* strain FA1090 is labelled. (**B**) The active site region of GalE from *E. coli* in complex with NADH and UDP-GlcNAc (PDB code 1LRJ)^17^. The protein alpha carbon trace is shown. The bound ligands and side chains for residues involved in interaction with the bound ligands are indicated by stick representation. A mesh surface is shown for Tyr299. (**C**) The active site region of GalE from *H. sapiens* in complex with NADH and UDP-GlcNAc (PDB code 1HZJ)^17^. The bound ligands and side chains for residues involved in interaction with the bound ligands are indicated by stick representation. A mesh surface is shown for Cys307. (**D**) The active site region of the PHYRE2 predicted structure of GalE from *N. meningitidis* MC58. The conserved side chains for residues implicated in interaction with the substrates are indicated by stick representation. A mesh surface is shown for Phe300. (**F**) The active site region of the PHYRE2 predicted structure of GalE from *N. gonorrhoeae* FA1090. The conserved side chains for residues implicated in interaction with the substrates are indicated by stick representation. A mesh surface is shown for Ser299.

**Figure 4:**
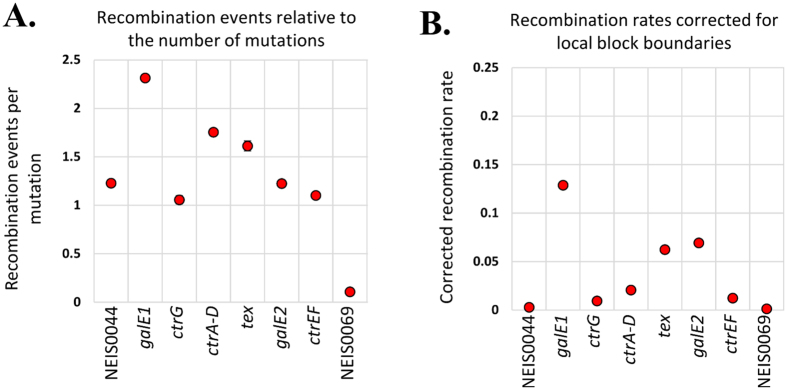
Recombination and mutation rates across the *cps* locus genes. Parsed ClonalFrame output detailing the origin of the rates of change for each locus. Panel A shows the relative rate of occurrence of recombination and mutation for each locus such that a value greater than 1.0 on the vertical axis (*ρ/θ*) indicates a higher frequency of recombination than mutation. Panel B shows the intensity of recombination for each locus as inferred by ClonalOrigin such that high values on the vertical axis (*ρ*_*s*_/*θ*_*s*_) indicate hotspots for recombination.

**Figure 5:**
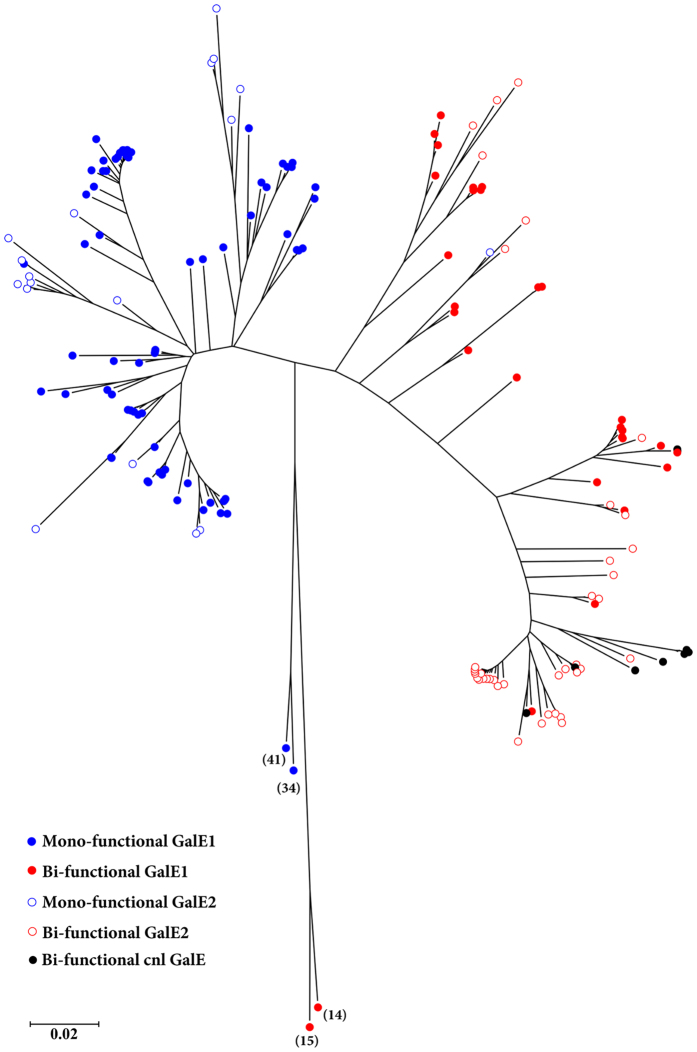
Phylogenetic tree of *galE1* and *galE2* alleles from *N. meningitidis*. The phylogenetic tree was constructed using the Neighbor-Joining method with 1000 bootstraps and evolutionary distances were calculated by the maximum composite likelihood method. In order to generate the tree, the alignment of the nucleotides was performed using MUSCLE after trimming the *galE1* and *galE2* alleles to the last 615 nucleotides. The colours indicate the functionality of the protein associated with the alleles; the filled and empty circles indicates *galE1* and *galE2* alleles respectively. The asterisks indicate *galE1* alleles (given in brackets) from *cnl* isolates.

Functional and phylogenetic analysis of *galE1* revealed that all *Neisseria* spp. possessed the gene, but that the functionality of the epimerase was predicted to vary across species (Fig. 2). Although the role of UDP-Gal and UDP-GalNAc is well described for the pathogenic *Neisseria* spp., very little is known regarding other species. Immunotyping studies have proposed that *N. cinerea, N. polysaccharea* and *N. lactamica,* which possess putative bi-functional *galE1* alleles, synthesise a LOS containing Gal and GalNAc residues but no structural studies have been done to confirm this^29^,^30^,^31^. The bi-functional meningococcal *galE1* alleles (Supplementary Fig. S2), were found in the presence of serogroup A, B, E, Y, I and K synthetic cassettes. A representative of this group, GalE_236 allele, had an equilibrium similar to that of the bi-functional allele of *N. gonorrhoeae*, thereby suggesting that the synthesis of UDP-GalNAc is the favoured outcome for this reaction (Table 1). This allele was found in *N. meningitidis* strain NMB which was shown to synthesise UDP-GalNAc by Lee *et al*.^32^. Although UDP-GalNAc is not present in LOS of this isolate^33^, it may be involved in another synthetic pathway such as protein glycosylation which has been recently detected in commensal *Neisseria* spp.^34^,^35^. However, since the GalNAc lipopolysaccharide transferase, *lgtD,* has been found in some isolates of meningococci, the capacity to add GalNAc to LOS could occur if these strains also possessed a bi-functional *galE1*^36^. The phylogeny of the meningococcal *galE* alleles (Fig. 5) suggests that bi-functional *galE1* alleles found in serogroups A/B/C/Y/W may have arisen sporadically through recombination with the *galE2* remanent or through point mutation of the active site of the *galE1* allele (Fig. 4). In contrast, the bi-functional *galE1*_14 allele found in serogroup E- expressing isolates is phylogenetically distant from these bi-functional *galE1* alleles (Fig. 5) suggesting a separate heritage potentially with the closest relative, *N. weaveri* (Fig. 1).

**Figure 6:**
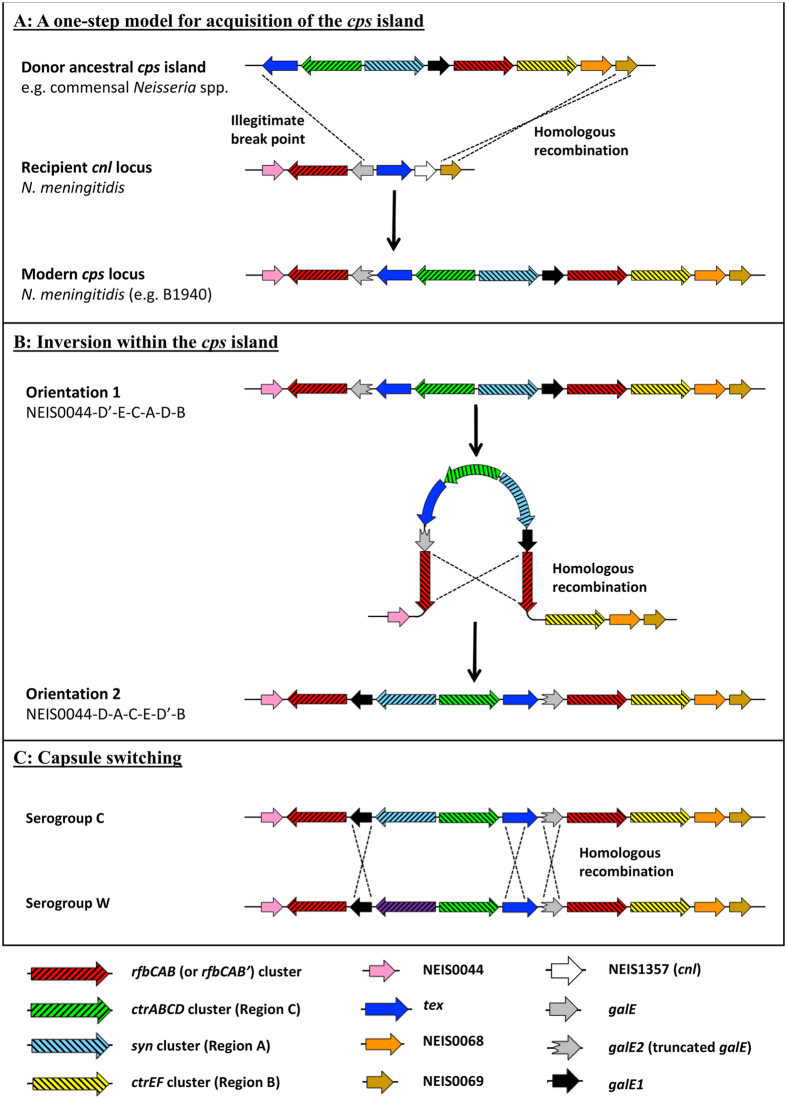
Proposed hypothetical model of events in the capture and re-organisation of the *cps* island in meningococci. (**A**) A hypothetical E-C-A-D-B cassette, containing a mono-functional *galE1*, was formed in an ancestral isolate (donor). This was recombined into a recipient strain containing a *cnl* locus to create the arrangement of the *cps* island found in modern pathogenic *N. meningitidis* in which the A-C-E cassette is flanked by the repetitive regions D and D’. (**B**) The D’-E-C-A-D cassette may undergo inversion via homologous recombination since Region D and D’ are able to form a hair-pin loop. (**C**) Serogroup switching involves the replacement of one serogroup synthetic cassette (eg. Serogroup C) with another (eg. Serogroup W). Multiple recombination events have been shown to occur within *galE1, tex* and *galE2* in multiple studies (see text).

The phylogenetic relationship of the bi-functional *galE2* alleles from pathogenic meningococci and the *galE* alleles in the *cnl* of non-pathogenic isolates led to the hypothesis that Region D’ was the original locus into which the *cps* island recombined (Fig. 6A). Since statistical association analysis demonstrated linkage disequilibrium between *galE1* and *galE2* as well as *galE1/galE2* pairs within each clonal complex, this suggested that the pairing of *galE1* with *galE2* occurred upon the acquisition of the *cps* island into the *cnl* locus of the ancestral strain of each clonal complex. One event that could result in this arrangement is the illegitimate recombination of an ancestral *cps* island consisting of Region A-C-E-D**-**B into *galE* on the left boundary and NEIS0069 on the right boundary of the recipient *cnl* locus (Fig. 6A). The strong associative properties of the *galE1/galE2* (Region A), *tex* (Region E) and *ctrEF* (Region B) alleles with clonal complex supports the concept of en bloc transfer and provides an explanation for the creation of Region D’ due to an illegitimate recombination event that truncates the *galE* allele of the *cnl* locus to become the remnant *galE2* locus (Fig. 6A).

The initial organisation of the *cps* locus predicted by this model is NEIS0044-region D’(*galE2*)- E- C- A- (*galE1*)D- B- NEIS0069 which is the same as that of the closed genome of strain B1940^23^. However, the closed genomes of strains MC58 and FAM18 are reported as NEIS0044-region D (*galE1*)- A- C- E- (*galE2*) D’- B- NEIS0069, suggesting that an inversion of the A-C-E block between region D and region D’ could occur. This was confirmed by directional PCR of the *cps* locus using plate grown strains MC58 and FAM18 (Supplementary Fig. S4). Since single colony PCR revealed that each colony contained a mixture of *cps* orientations (data not shown), it appears that this process is dynamic, occurring during chromosomal replication when single stranded DNA is present for RecA mediated homologous recombination (Fig. 6B). Unless the duplicated Region D’ is deleted, the locus is not able to be fixed in any particular orientation. Colony immunoblots of strain NMB which is not phase variable for capsule expression did not detect any mixed phenotypes consistent with previous studies on the expression of capsule by this strain (data not shown^37^,^38^,^39^). It is interesting to note that the *cps* island is inserted 54 kb downstream of the origin of replication which is surrounded by a number of genomic inversions^40^. In *E. coli*, Ivanova *et al*.^41^ have recently shown that inversions surrounding the origin of replication occur to resolve collisions between highly transcribed genes and the direction of chromosomal replication, thus improving fitness. It is currently unclear whether this mechanism applies to this situation in *N. meningitidis* but is worth considering in future investigations of this region.

The proposed en bloc model for the transfer of the *cps* island into meningococcal clonal complexes relies upon the concept previously advanced by Schoen *et al*.^6^,^7^,^42^ that the mosaic structure is created in an ancestral meningococcal isolate. Recently, Harrison *et al* (personal communication) have detected similar *cps* islands consisting of regions A-C-E-D-B, and lacking Region D’, in commensal *Neisseria* spp. which could also be a source of the donor element for the en bloc transfer hypothesis. Currently the PubMLST database does not contain meningococcal isolates lacking Region D’ as the majority of isolates represented in this collection have been isolated from cases of disease. It will be interesting to examine a larger collection of non-disease causing meningococcal isolates for the presence of strains bearing incomplete *cps* islands as these may provide further evidence for the model. This model was also used to examine serogroup switching, which from the observations of Mustapha *et al*.^12^ disrupt the linkage disequilibrium of *galE1* and *galE2*. An examination of other serogroup switching events in the database indicated that this was a general phenomenon associated with this HGT event.

In conclusion, we propose evidence for a hypothetical model in which the acquisition of the *cps* island via HGT results in the loss of UDP-GalNAc synthesis from UDP-GlcNAc in most pathogenic meningococcal isolates, except for those expressing serogroup E capsules. UDP-GlcNAc is an entry metabolite for the synthesis of cell wall components, peptidoglycan and lipid A, in addition to sialic acid found in serogroup B, C, W and Y capsular types. Presumptively, the inactivation of the bi-functional *galE* allele of the *cnl* locus and replacement with a mono-functional *galE1* allele releases a pool of UDP-GlcNAc that can be redirected into the sialic acid capsule biosynthesis pathway while minimising the metabolic fitness cost associated with the acquisition of the *cps* island in pathogenic meningococci. However, other accessory changes to central metabolism are likely to be required. Mustapha *et al*.^12^ noted that the capsule switching event in cc11 from serogroup C to W included central metabolic genes such as the pyruvate kinase. In addition, Schoen *et al*.^43^ found that the central metabolism of pathogenic meningococci is diverse and that multiple adaptive changes to metabolism have occurred thus providing a framework in which to test our theory regarding the fitness costs associated with the acquisition of the *cps* synthesis pathway.

## Materials and Methods

### Bacterial strains and growth conditions

Meningococcal strains were cultured under aerobic conditions with 5% CO_2_ at 37 °C on GC agar (GCA) or GC broth (GCB) (Oxoid) supplemented with 0.4% glucose, 0.01% glutamine, 0.2 mg of cocarboxylase per litre, and 5 mg of Fe(NO_3_)_3_ per litre. The wild-type strains and constructed mutants used in this study are shown in Supplementary Table S5. Antibiotic selection for meningococcal mutants was performed on GCA containing 100 μg/ml of kanamycin (sulfate salt), 60 μg/ml of spectinomycin, 5 μg/ml of tetracycline or 2 μg/ml of erythromycin (Sigma). *Escherichia coli* DH5α was used as a host for all DNA manipulations and Rosetta™ (DE3) as a host for all protein expression. *E. coli* strains were routinely grown on Luria-Bertani broth (LBB) and agar (LBA, Oxoid) which, where appropriate, was supplemented with antibiotics at the following concentrations: ampicillin at 100 μg/ml, spectinomycin at 50 μg/ml, kanamycin at 50 μg/ml, erythromycin at 300 μg/ml, tetracycline at 12.5 μg/ml and chloramphenicol at 30 μg/ml (Sigma).

### Construction of expression strains

The plasmids constructed and used in this study are listed in Supplementary Table S5. Genomic DNA was isolated from *N. gonorrhoeae* strain FA1090 and *N. meningitidis* strains NMB and MC58 using the Purelink^TM^ genomic DNA purification kit (Invitrogen) according to manufacturer’s instructions. The gene *galE* was amplified with the primers KAP580 (5′-GGAATTCCATATGAAAAAAATTCTCGTTACCG-3′) and KAP581 (5′-CGCGGATCCTTAATCGTCGTAGCCATTCGG-3′) for MC58, KAP582 (5′-GGAATTCCATATGACCGTCCTGATTACCG-3′) and KAP583 (5′-CGCGGATCCTTAATCCCCATATCTGCCG-3′) for FA1090 and KAP563 (5′GGAATTCCATATGCCCTATACGGAAGATATG-3′) and KAP564 (5′-CGCGGATCCTTAATCCCCATATCCGTTGGG-3′) for NMB, such that there was a 5′ *Nde*I site overlapping the start codon and a 3′ *Bam*HI site downstream of the stop codon incorporated into the PCR fragment. Each PCR product was directionally cloned into the *Nde*I and *Bam*HI sites of pET15b, resulting in the 5′ fusion of the open reading frame with a hexahistidine motif, resulting in plasmids pCMK730, pCMK729 and pCMK771. The *galE* of *N. gonorrhoeae* and *N. meningitidis* that have been cloned into pET15b were sequenced and their sequence found to be identical to the native gene.

The cloned *galE* of *N. gonorrhoeae* strain FA1090 and *N. meningitidis* strain MC58 were mutagenised using site directed mutagenesis to change a single nucleotide. The primers KAP427 (5′-CGACTTGGCGTGTTTCTATGCCGACCC-3′) and KAP428 (5′-GGGTCGGCATAGAAACACGCCAAGTCG-3′) to mutate the gonococcal *galE*, and KAP429 (5′-GGTGATTTGGCGTGCTCCTATGCCGACCC-3′) and KAP430 (5′-GGGTCGGCATAGGAGCACGCCAAATCACC-3′) to mutate the meningococcal *galE* were used to amplify the plasmids pCMK729 and pCMK730 respectively using the non-strand displacing polymerase Phusion (New England Biolabs). The template DNA was digested with *Dpn*I, prior to transformation into DH5α. The resulting plasmids were sequenced and confirmed to contain only the introduced mutation. These plasmids were called pCMK733 and pCMK734 respectively.

Expression strains were constructed by transforming the plasmids pCMK729, pCMK730, pCMK771, pCMK733 and pCMK734 into BL21-DE3 Rosetta. Inducible protein expression from these strains were confirmed by growing the strains to mid-log phase in 10 ml LBB, splitting the culture in two, and inducing one culture with 0.3 mM IPTG for 1 hr. The cells from 1 ml of each culture was collected by centrifugation and resuspended in 100 μl of distilled water to which 25 μl of 5x Laemelli loading buffer was added. The samples were boiled for 10 mins prior to separation on 12% SDS-PAGE at 150 V, then stained with Coomassie Blue R250. Expression was determined by the presence of a band of increased intensity present in the induced sample compared to the un-induced sample at the correct molecular weight.

Expression of His-tagged GalE was confirmed by Western immunoblot. Whole cell lysates (750 ng) were separated by 15% sodium dodecyl sulfate-polyacrylamide gel electrophoresis (SDS-PAGE) by standard methods and transferred to nitrocellulose membranes. The membranes were blocked overnight with 2% BSA in TBS, then incubated with the monoclonal mouse anti-His IgG (Sigma) primary antibody at 1:1,000 dilution. Horse radish peroxidase-conjugated anti-Rabbit IgG and anti-Mouse IgG secondary antibodies (Santa Cruz Biotechnology) was used for detection and the membrane was developed with an ECL kit (GE Healthcare).

### Protein purification

*E. coli* strains expressing meningococcal GalE proteins (both native and mutagenised) were grown overnight with shaking in 10 ml LBB containing 100μg/ml ampicillin. An aliquot of 100μl was used to inoculate 10 ml LBB containing 100 μg/ml ampicillin the following day and the strains grown to OD_600_ 0.6–0.8 at which time protein expression was induced by the addition of IPTG to a final concentration of 0.3 mM. Induction occurred for 3 hr at 37 °C prior to the purification of protein using the Wizard HisLink^TM^ Spin Protein Purification System (Promega) as per manufacturer’s instructions. The gonococcal GalE could not be purified in this way due to the formation of inclusion bodies. Strains expressing the gonococcal GalE proteins were grown overnight with shaking in 10 ml LBB containing 100μg/ml Ampicillin. An aliquot of 1 ml was used to inoculate 1 L LBB containing 100μg/ml Ampicillin the following day and the strains grown to OD_600_ 0.6–0.8 at which time protein expression was induced by the addition of IPTG to a final concentration of 0.3 mM. Induction occurred for 3 hr at 37 °C prior to the collection of all bacterial cells by centrifugation at 3000 rcf for 15 mins at 4 °C. The pellet was resuspended in 50 ml binding buffer (20 mM sodium phosphate, 0.5 M NaCl and 20 mM imidazole, pH 7.4) and sonicated 1 min on, 1 min off for 40 mins. Cell debris was removed by centrifugation and the supernatant applied to a HisTrap^TM^ FF column. The column was washed with 10 volumes of binding buffer and the protein eluted in 10 volumes of elution buffer (20 mM sodium phosphate, 0.5 M NaCl and 500 mM imidazole, pH 7.4).

The purified proteins were dialysed against 100 volumes of dialysis buffer (10% glycerol, 20 mM Tris/HCl, pH 7.9), changed three times. The gonococcal GalE proteins were concentrated to 100 μl using a centricon-10 (Millipore, Germany). Protein concentrations were determined by Bradford assay and glycerol was added to a final concentration of 50% (v/v) to stabilize the enzyme.

### High Performance Liquid Chromotography

Activity of the gonococcal and meningococcal GalE proteins, and the site directed mutagenised proteins was done using methods described in Dong *et al*.^44^. Briefly, the activated sugar substrates (UDP-Glc, UDP-Gal, UDP-GlcNAc and UDP-GalNAc, 1 mM) were incubated with 200 ng of purified protein in 20 mM Tris-HCl, 4 mM Mg^2+^, and 1 mM NAD^+^ (pH 8.0) in a final volume of 50 μl. The reactions were performed at 37 °C for 2 h and were terminated by heating at 100 °C for 5 min. The products from the reaction were run on HPLC using a UV detector and compared to UDP-Glc, UDP-Gal, UDP-GlcNAc and UDP-GalNAc standards.

### Phylogenetic analysis of *galE1* and *galE2* alleles from *N. meningitidis*

A dataset of 194 isolates of *Neisseria* spp. was selected from the Bacterial Isolate Genome Sequence Database (BIGSDB) located at PubMLST (http:pubmlst.org/neisseria)^45^ and described in Supplementary Table S1. This dataset was selected to include the most common alleles of and provide the maximal diversity for NEIS0048 and NEISS0062 across 133 *N. meningitidis* isolates, 16 *N. gonorrhoeae* isolates, 17 *N. lactamica* isolates, seven *N. cinerea* isolates, six *N. polysaccharea* isolates, four *N. oralis* isolates^42^,^46^, four *N. elongata* isolates, four *N. subflava* isolates and one isolate each from the *Neisseria* species *N. bergeri, N. animalis* and *N. weaveri.* All of these genomes were sequenced on an Illumina Genome Analyser II platform and assembled according to Jolley and Maiden^45^. However, because Region D and D’ are duplicated regions, each contig was manually checked and exported as XMFA files containing aligned sequence blocks and then converted to a fastA format for import into MEGA version 5.0^47^ and Splitstree^48^.

The *galE1* and *galE2* alleles were downloaded from the *Neisseria* PubMLST database (http://pubmlst.org/neisseria/)^45^ as NEIS0048 and NEIS0062 respectively. The phylogeny of the GalE1 peptide sequences was reconstructed using the neighbor-joining method using the MEGA software package^47^ with the following parameters: Bootstrap (1000 replicates), using a Poisson model and gamma distribution (parameter set to 1). For the phylogeny of the conserved regions of the *galE1* and *galE2* alleles, the sequences were trimmed using UGENE and the final 615 nucleotides were aligned using MUSCLE. Phylogenetic analysis was conducted by the neighbor-joining method using 1000 bootstrap tests. Evolutionary distances were calculated by the maximum composite likelihood method. The analysis was conducted with MEGA6.06.

### Clonal Frame analysis of recombination relative to mutation

The distribution of NEIS0048 and NEIS0062 was assessed in 1196 pathogenic meningococcal isolates summarised in Supplementary Table S2. Aligned sequences were analysed using the default parameters of ClonalFrame^49^ and the output parsed using the ParseCF script written by Barry Hall. Values calculated by ClonalFrame and ParseCF include the mutation rate (θ), the mutation rate per site (θ/number of sites), the corrected recombination rate for local block boundaries (ρ/number of sites), the estimate of recombination relative to that of mutation (r/m) and the estimate of the number of recombination events relative to mutations (ρ/θ).

### PCR amplification of *cps* island inversion

KAP727 (5′-GAAGAATACCGCGAACTGACGC-3′) is a forward primer specific to the 5′ end of NEIS0044; KAP730 (5′-AGTTGGAAGAGCGCAAAGCCG-3′) is a forward primer specific to the 5′ end of *tex* (NEIS0059); KAP735 (5′-TTCAGACGGCAAGAGGGTACG-3′) is a reverse primer specific to the 3′ end of *ctrE* (NEIS0066). The PCR was performed using Phusion^®^ High-Fidelity DNA Polymerase (New England Biolabs). PCR conditions for the assay were 2 min at 98 °C followed by 35 cycles of 98 °C for 10 seconds, 65 °C for 30 seconds and 72 °C for 10 minutes with a final extension time of 10 minutes at 72 °C.

## Additional Information

**How to cite this article**: Bartley, S. N. *et al*. Acquisition of the capsule locus by horizontal gene transfer in Neisseria meningitidis is often accompanied by the loss of UDP-GalNAc synthesis. *Sci. Rep.* **7**, 44442; doi: 10.1038/srep44442 (2017).

**Publisher's note:** Springer Nature remains neutral with regard to jurisdictional claims in published maps and institutional affiliations.

## Supplementary Material

Supplementary Information

## Figures and Tables

**Table 1:** Analysis of UDP-Glucose and UDP-GlcNAc 4-epimerase activity of GalE alleles and their mutants.

Enzyme	Substrate	Equilibrium ratio UDP-Glc:UDP-Gal	Equilibrium ratio UDP-GlcNAc:UDP-GalNAc
Gonococcal GalE_17	UDP-Glucose	39:61	—
	UDP-GlcNAc	—	32:68
GalE_17 (S299F)	UDP-Glucose	59:41	—
	UDP-GlcNAc	—	99.2:0.8
Meningococcal GalE_2	UDP-Glucose	63:37	—
	UDP-GlcNAc	—	99.5:0.5
GalE_2 (F300S)	UDP-Glucose	67:33	—
	UDP-GlcNAc	—	73:27
GalE_236	UDP-Glucose	23:77	—
	UDP-GlcNAc	—	25:75
