# Supplementary material for: Acquisition of the capsule locus by horizontal gene transfer in Neisseria meningitidis is often accompanied by the loss of UDP-GalNAc synthesis
Source: Sci Rep. 2017 Mar 14;7:44442. doi: 10.1038/srep44442 (PMC5349592; doi:10.1038/srep44442)
Supplement: Supplementary Information [file srep44442-s1.pdf]

Acquisition of the capsule locus by horizontal gene transfer in *Neisseria meningitidis* is often accompanied by the loss of UDP-GalNAc synthesis

(Supplementary Materials)

Stephanie N. Bartley*[1,2], Shakeel Mowlaboccus*[1], Christopher A. Mullally[1], Keith A. Stubbs[2], Alice Vrielink[2], Martin C. J. Maiden[3], Odile B. Harrison[3], Timothy T. Perkins[1], and Charlene M. Kahler[1,§]

[1]School of Pathology and Laboratory Medicine, and the Marshall Centre for Infectious Disease Research and Training, University of Western Australia, Perth, Australia.

[2]School of Chemistry and Biochemistry, University of Western Australia, Perth, Australia

[3]University of Oxford, Department of Zoology, South Parks Road, Oxford OX1 3PS, United Kingdom

*Co-First authors

[§]Corresponding author, email: charlene.kahler@uwa.edu.au

```
H.sapiens         MAEKVLVTGGAGYIGSHTVLELLEAGYLPVVIDNFHNAFRGGGSLPESLRRVQELTGRSV 60
Y.enterolytica    --MSILITGGAGYIGSHTVLTLLEQGRNVVVLDNLIN------SSAESLARVSKICGRKP 52
N.gonorrhoeae     --MTVLITGGTGFIGSHTAVSLVQSGYDAVILDNLCN------SSAAVLPRLRQITGRNI 52
N.meningitidis    -MKKILVTGGTGFIGSHTVVSLLKSGHQVVILDNLCN------SSINILPRLKTITGQEI 53
E.coli            --MRVLVTGGSGYIGSHTCVQLLQNGHDVIILDNLCN------SKRSVLPVIERLGGKHP 52
                      :*:***:*:***** : *:: *   :::**: *       *    *  :  : *:

H.sapiens         EFEEMDILDQGALQRLFKKYSFMAVIHFAGLKAVGESVQKPLDYYRVNLTGTIQLLEIMK 120
Y.enterolytica    NFYHGDILDRSCLKLIFSSHKIDSVIHFAGLKSVGESVEKPIEYYQNNVVGSITLLEEMC 112
N.gonorrhoeae     PFYQGDIRDCQILRQIFSEHEIESVIHFAGLKAVGESVAEPTKYYGNNVYGSLVLAEEMA 112
N.meningitidis    PFYQGDIRDREILRRIFAENRIDSVIHFAGLKAVGESVAEPMKYYDNNVSGSLVLAEEMA 113
E.coli            TFVEGDIRNEALMTEILHDHAIDTVIHFAGLKAVGESVQKPLEYYDNNVNGTLRLISAMR 112
                   * . ** :   :  :: .  : :********:***** :* .**  *: *:: * . *

H.sapiens         AHGVKNLVFSSSATVYGNPQYLPLDEAHPTGGCTNPYGKSKFFIEEMIRDLCQADKTWNA 180
Y.enterolytica    LANVKKLIFSSSATVYGEPEFVPLTENARIGGTTNPYGTSKVMVEQILKDFSLAHPDYSI 172
N.gonorrhoeae     RAGVLKIVFSSSATVYGDAEKVPYTEDMRPGDTANPYGASKAMVERMLTDIQKADPRWSV 172
N.meningitidis    RAGVFSIVFSSSATVYGDPGKVPYTEDMPPGDTTSPYGASKSMVERILTDIQKADPRWSM 173
E.coli            AANVKNFIFSSSATVYGDQPKIPYVESFPTGTPQSPYGKSKLMVEQILTDLQKAQPDWSI 172
                    .*  .::*********:   :*  *    *    .*** ** ::*.:: *:  *.  :.

H.sapiens         VLLRYFNPTGAHASGCIGEDPQGIPNNLMPYVSQVAIGRREALNVFGNDYDTEDGTGVRD 240
Y.enterolytica    TALRYFNPVGAHPSGLIGEDPNGKPNNLLPFITQVAIGKLSKLLVYGNDYDTPDGSGIRD 232
N.gonorrhoeae     ILLRYFNPIGAHESGLIGEQPNGVPNNLLPYICQVASGRLPQLSVFGGDYPTPDGTGMRD 232
N.meningitidis    ILLRYFNPIGAHESGLIGEQPNGIPNNLLPYICQVAAGKLPQLAVFGDDYPTPDGTGMRD 233
E.coli            ALLRYFNPVGAHPSGDMGEDPQGIPNNLMPYIAQVAVGRRDSLAIFGNDYPTEDGTGVRD 232
                    ****** *** **  :**:*:* ****:*:: *** *:    *  ::*.** * **:*:**

H.sapiens         YIHVVDLAKGHIAALRKLKEQCGCRIYNLGTGTGYSVLQMVQAMEKASGKKIPYKVVARR 300
Y.enterolytica    YIHVMDLAEGHLSTLINLTS--GFRIYNLGTGVGYSVLHMIKEFERITGKNIPFDIVSRR 290
N.gonorrhoeae     YIHVMDLAEGHIAAMKAKGGVAGVHLFNLGSGRAYSVLEIIRAFEAASGLHIPYRIQPRR 292
N.meningitidis    YIHVMDLAEGHVAAMQAKSNVAGTHLLNLGSGRASSVLEIIRAFEAASGLTIPYEVKPRR 293
E.coli            YIHVMDLADGHVVAMEKLANKPGVHIYNLGAGVGNSVLDVVNAFSKACGKPVNYHFAPRR 292
                  ****:***.**: ::       * :: ***:* . ***.::. :.   *  : : . .**

H.sapiens         EGDVAACYANPSLAQEELGWTAALGLDRMCEDLWRWQKQNPSGFGTQA 348
Y.enterolytica    PGDIAECWASPELAHLELGWYAKRTLVDMLQDAWKWQKMNPNGYNC-- 336
N.gonorrhoeae     AGDLACSYADPSHTKQQTGWETKRGLQQMMEDSWRWVSRNPGRYGD-- 338
N.meningitidis    AGDLACFYADPSYTKAQIGWQTQRDLTQMMEDSWRWVSNNPNGYDD-- 339
E.coli            EGDLPAYWADASKADRELNWRVTRTLDEMAQDTWHWQSRHPQGYPD-- 338
                   **:.   :*... :. : .* .    *   *  :* *:* . :*   :
```

Supplementary Figure S1. Alignment of mono-functional and bi-functional GalE sequences. The protein sequences of the known bi-functional GalE of *Y. enterolytica* (18), the bi-functional GalE from *Homo sapiens* (17), the known mono-functional GalE sequence of *E. coli* (17) were aligned with the GalE sequences of *N. gonorrhoeae* strain FA1090 and *N. meningitidis* strain MC58 using ClustalW2. The amino acids previously demonstrated to be associated with mono- and bi-functionality are indicated in grey (17). The variable amino acids in the active site are shown in red. The consensus line is as follows: "*", identical residues, ":" conservative replacement, "." semi-conserved.

Supplementary Figure 2

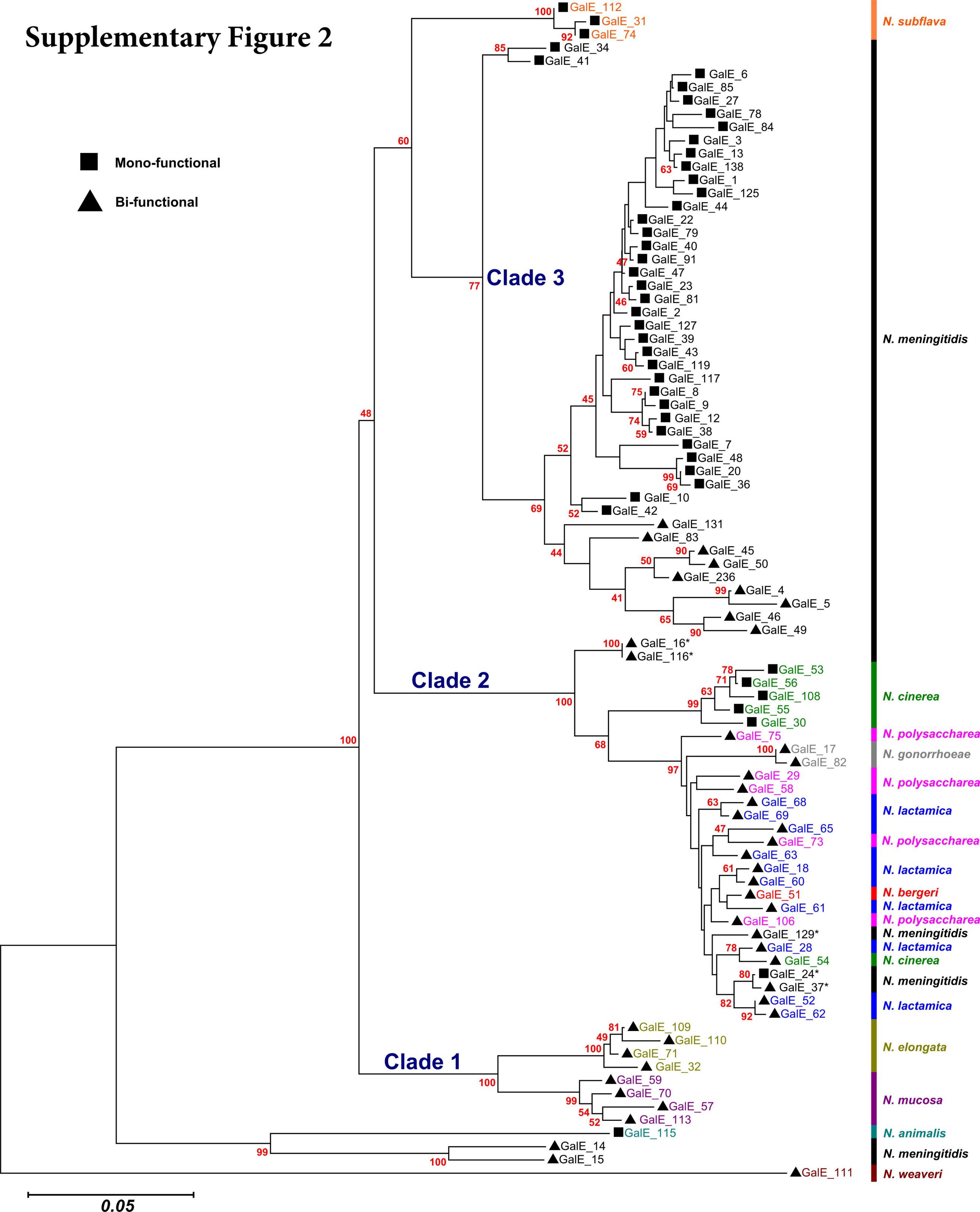

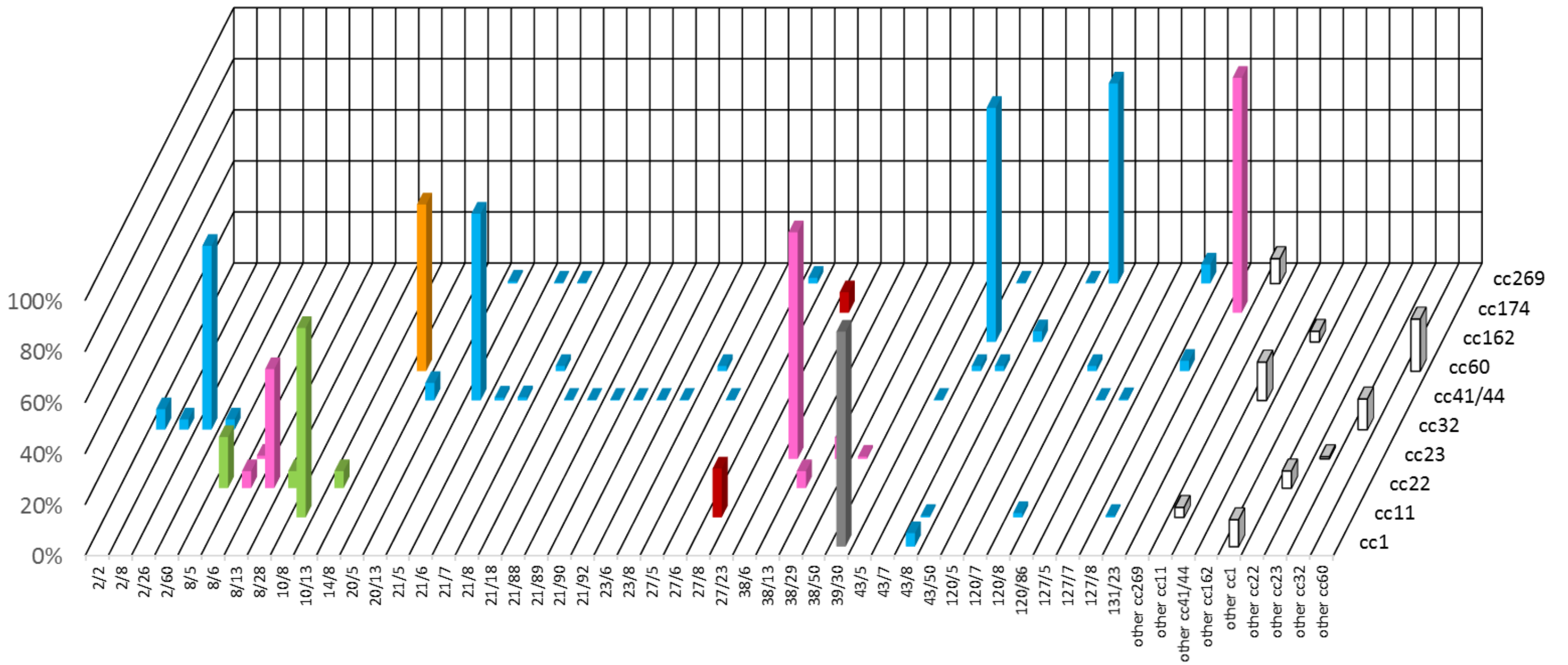

Supplementary Figure S3. Linkage disequilibrium of GalE1/GalE2 allelic pairs associated with different serogroups within clonal complexes.

The percentage of strains (y-axis) possessing the GalE1/GalE2 allelic pair (x-axis) was plotted for ten clonal complexes (z-axis). Each bar is coloured according to serogroup (Blue = serogroup B, Red = serogroup C, Orange = serogroup E, Grey= serogroup A, Green = serogroup W, Pink = serogroup Y). The category "other cc_x" corresponds to GalE1/GalE2 allelic pairs identified in isolates belonging to cc_x for which serogrouping data was not available.

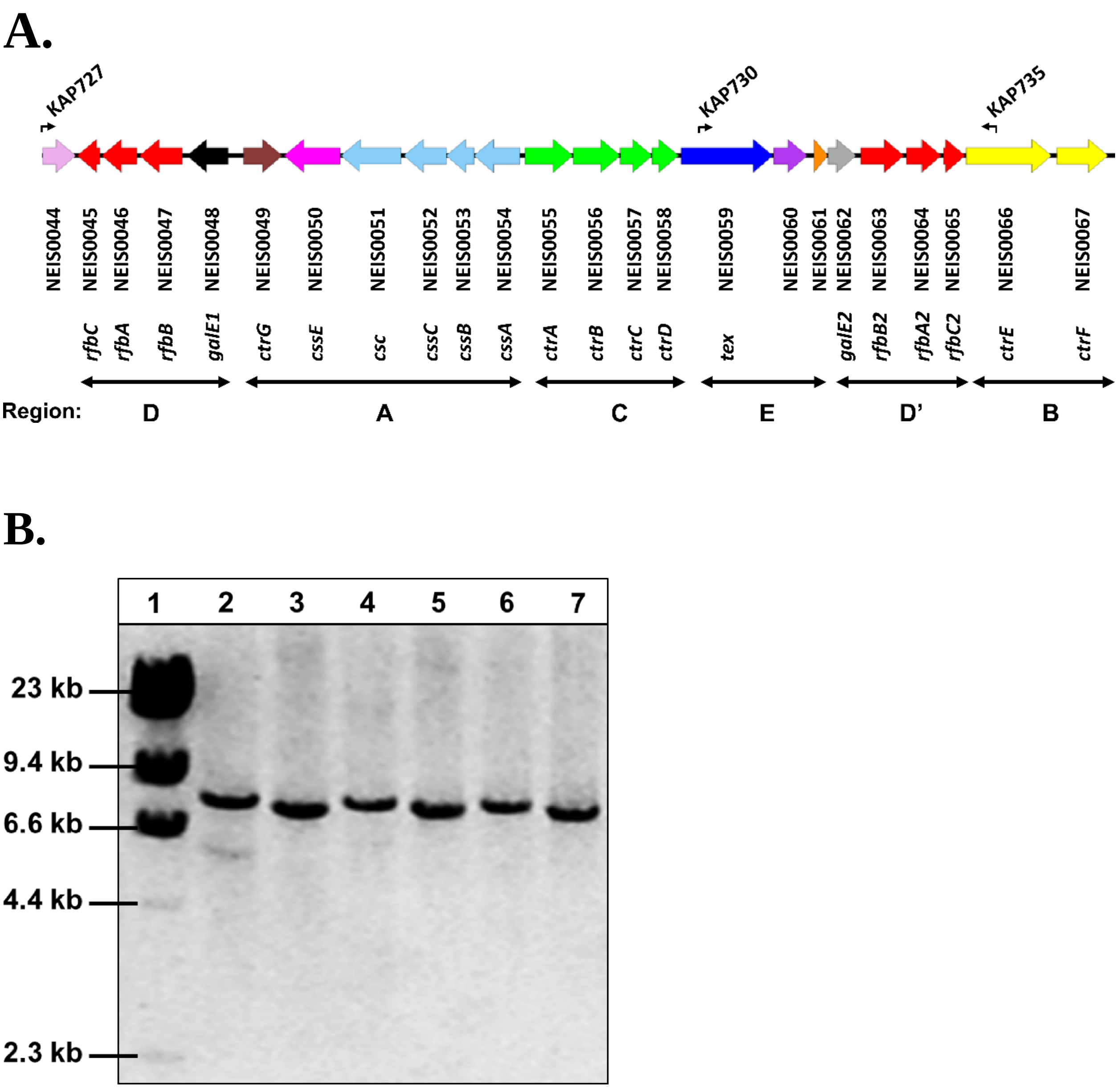

Supplementary Figure S4. Inversion of region A-C-E at the *cps* locus detected in NMB (B:cc8), MC58 (B:cc32) and FAM18 (C:cc11).

A PCR assay for the detection of inversion within region D-A-C-E-D' to form D-E-C-A-D' (panel A) was performed with the primer pair KAP727 and KAP730, which generates an 8.9 kb amplicon when there is inversion such that NEIS0044 and region E are anti-parallel (lanes 2, 4 and 6). An 8.0 kb amplicon is generated using the primer pair KAP730 and KAP735 when NEIS0044 and region E are parallel (lanes 3, 5 and 7). Both orientations were detected in strain NMB (lanes 2 and 3), strain MC58 (lanes 4 and 5) and strain FAM18 (lanes 6 and 7) showing that inversion of the block of genes occurs *in-vivo* (panel B).

Supplementary Table S1. ***Neisseria spp.*** **used in this study.**

Isolate	Disease	Serogroup	*galE1* allele	Residue at amino acid 300	Bi-functional/ Mono-functional	Accession number & references
N. meningitidis						
120M	invasive	A	39	F	mono-functional	This publication
6748	invasive	A	39	F	mono-functional	This publication
20	invasive	A	39	F	mono-functional	This publication
393	carrier	A	39	F	mono-functional	This publication
S5611	invasive	A	40	F	mono-functional	This publication
BZ 133	invasive	C	43	F	mono-functional	This publication
79128	invasive	A	79	F	mono-functional	This publication
322/85	invasive	A	39	F	mono-functional	This publication
79126	invasive	A	50	S	bi-functional	This publication
129E	invasive	A	39	F	mono-functional	This publication
254	invasive	A	39	F	mono-functional	This publication
371	invasive	A	39	F	mono-functional	This publication
106	invasive	A	39	F	mono-functional	This publication
CN100	invasive	A	39	F	mono-functional	This publication
2059001	invasive	A	34	F	mono-functional	This publication
Z2491	meningitis	A	13	F	mono-functional	This publication
WUE 2594	meningitis	A	81	F	mono-functional	FR774048 (10)
BZ 10	invasive	B	138	F	mono-functional	This publication
BZ 163	invasive	B	139	F	mono-functional	This publication
G2136	invasive	B	144	S	bi-functional	CP002419 (48)
B6116/77	invasive	B	140	S	bi-functional	This publication
94/155	invasive	C	141	S	bi-functional	This publication
312 901	invasive	C	142	S	bi-functional	This publication
AK22	invasive	B	143	S	bi-functional	This publication
SB 25	invasive	C	141	S	bi-functional	This publication

961-5945	unspecified	B	126	S	bi-functional	AEQK01000076 (48)
FAM18	invasive	C	1	F	mono-functional	NC008767 (39)
500	unknown	C	1	F	mono-functional	This publication
38VI	unknown	B	42	F	mono-functional	This publication
M597	invasive	C	77	F	mono-functional	This publication
NG P20	invasive	B	1	F	mono-functional	This publication
BRAZ10	unknown	C	42	F	mono-functional	This publication
2839	invasive	C	27	F	mono-functional	(49)
2838	carrier	C	27	F	mono-functional	(49)
M6190	invasive	B	23	F	mono-functional	AEQF01000000 (48)
ES14902	invasive	B	27	F	mono-functional	AEQI01000000 (48)
MC58	invasive	B	2	F	mono-functional	NC003112 (50)
H44/76	invasive	B	2	F	mono-functional	CP002420 (48)
196/87	unknown	C	41	F	mono-functional	This publication
NG 080	invasive	B	2	F	mono-functional	This publication
NG 144/82	invasive	B	2	F	mono-functional	This publication
NG PB24	invasive	B	2	F	mono-functional	This publication
CU385	invasive	B	2	F	mono-functional	AEQJ01000000 (48)
053442	invasive	C	3	F	mono-functional	NC010120 (51)
BZ 147	invasive	B	44	F	mono-functional	This publication
BZ 198	invasive	B	21	F	mono-functional	This publication
NG H15	carrier	B	21	F	mono-functional	This publication
NG E30	carrier	B	21	F	mono-functional	This publication
NG H36	carrier	B	21	F	mono-functional	This publication
400	invasive	B	36	F	mono-functional	This publication
AK50	invasive	B	21	F	mono-functional	This publication
50/94	invasive	B	21	F	mono-functional	This publication
M40/94	invasive	B	21	F	mono-functional	This publication
931905	invasive	B	21	F	mono-functional	This publication

N45/96	invasive	B	21	F	mono-functional	This publication
91/40	invasive	B	21	F	mono-functional	This publication
88/03415	invasive	B	21	F	mono-functional	This publication
NZ05/33	invasive	B	21	F	mono-functional	CP002424 (48)
M0579	invasive	B	21	F	mono-functional	AEQH01000000 (48)
M01.240149	invasive	B	21	F	mono-functional	CP002421 (48)
NG 6/88	invasive	B	21	F	mono-functional	This publication
NG F26	carrier	B	48	F	mono-functional	This publication
M04.240196	invasive	B	120	F	mono-functional	CP002423 (48)
M13399	invasive	B	125	F	mono-functional	AEQG01000000 (48)
M01.240013	invasive	B	124	F	mono-functional	AEQL01000000 (48)
BZ 232	invasive	B	45	S	bi-functional	This publication
NG 4/88	invasive	B	23	F	mono-functional	This publication
NG H41	carrier	B	78	F	mono-functional	This publication
NG E31	carrier	B	49	S	bi-functional	This publication
297-0	carrier	E	14	C	bi-functional	This publication
DK24	invasive	B	46	S	bi-functional	This publication
NG 3/88	invasive	B	47	F	mono-functional	This publication
NG G40	carrier	B	83	S	bi-functional	This publication
3906	invasive	B	35	F	mono-functional	This publication
29013	carrier	C	6	F	mono-functional	(18)
M01.240355	unspecified	B	127	F	mono-functional	CP002422 (48)
E26	carrier	*cnl*	37	S	bi-functional	This publication
alpha14	carrier	*cnl*	16	S	bi-functional	AM889136 (6)
2848	invasive	*cnl*	116	S	bi-functional	(49)
A22	carrier	W	8	F	mono-functional	This publication
WUE171	unspecified	W	10	F	mono-functional	2838(18)
71/94	invasive	Y	38	F	mono-functional	This publication

860800	invasive	Y	38	F	mono-functional	This publication
WUE172	carrier	Y	10	F	mono-functional	(18)
alpha162	carrier	Y	9	F	mono-functional	(18)
29031	carrier	H	12	F	mono-functional	(18)
29046	carrier	K	5	S	bi-functional	(18)
29043	carrier	I	4	S	bi-functional	(18)
WUE173	carrier	Z	15	C	bi-functional	(18)
WUE3608		L	7	F	mono-functional	(18)
M11 240474	invasive	C	22	F	mono-functional	This publication
BB238	unspecified	*cnl*	24	F	mono-functional	This publication
BB291	unspecified	*cnl*	24	F	mono-functional	This publication
R291	unspecified	*cnl*	24	F	mono-functional	This publication
R304	unspecified	*cnl*	24	F	mono-functional	This publication
T291	unspecified	*cnl*	24	F	mono-functional	This publication
2445	unspecified	B	84	F	mono-functional	This publication
79694	unspecified	B	84	F	mono-functional	This publication
93-N213	unspecified	NG	85	F	mono-functional	This publication
12026_2011	invasive	B	85	F	mono-functional	This publication
M11 240324	invasive	C	86	F	mono-functional	This publication
N24/99	unspecified	C	86	F	mono-functional	This publication
M11 240488	invasive	B	91	F	mono-functional	This publication
N1568	unspecified	ND*	117	F	mono-functional	This publication
M11 240048	invasive	B	119	F	mono-functional	This publication
V268	unspecified	ND*	119	F	mono-functional	This publication
BB89	unspecified	ND*	129	S	bi-functional	This publication
BB185	unspecified	*cnl*	129	S	bi-functional	This publication
R89	unspecified	ND*	129	S	bi-functional	This publication
90142v2	unspecified	*cnl*	129	S	bi-functional	This publication
90142v3	unspecified	*cnl*	129	S	bi-functional	This publication

M10 240759	invasive	Y	131	S	bi-functional	This publication
M11 240073	invasive	Y	131	S	bi-functional	This publication
M11 240161	invasive	Y	131	S	bi-functional	This publication
M11 240209	invasive	Y	131	S	bi-functional	This publication
BB60	unspecified	ND*	131	S	bi-functional	This publication
12006_2013	invasive	Y	131	S	bi-functional	This publication
NMB	invasive	B	236	S	bi-functional	This publication
M10 240747	invasive	B	20	F	mono-functional	This publication
M10 240823	invasive	B	20	F	mono-functional	This publication
M11 240003	invasive	B	20	F	mono-functional	This publication
M11 240024	invasive	B	20	F	mono-functional	This publication
M11 240193	invasive	B	20	F	mono-functional	This publication
M11 240360	invasive	B	20	F	mono-functional	This publication
M11 240476	invasive	B	20	F	mono-functional	This publication
M11 240707	invasive	B	20	F	mono-functional	This publication
M11 240728	invasive	B	20	F	mono-functional	This publication
M11 240736	invasive	B	20	F	mono-functional	This publication
BB77	unspecified	ND*	20	F	mono-functional	This publication
z77	unspecified	ND*	20	F	mono-functional	This publication
BB44	unspecified	ND*	20	F	mono-functional	This publication
12012_2012	invasive	B	20	F	mono-functional	This publication
NM3687	Invasive	W	10	F	mono-functional	This publication
N. lactamica						
020-06	carrier	N/A*	18	S	bi-functional	NC014752 (25)
ATCC23970	carrier	N/A*	28	S	bi-functional	ACEQ02000000
224	carrier	N/A*	60	S	bi-functional	(52) (53)
4116	carrier	N/A*	61	S	bi-functional	(52) (53)
09002S1	carrier	N/A*	62	S	bi-functional	(52) (27, 53)
8206	carrier	N/A*	61	S	bi-functional	(52) (53) (27)

005-12	carrier	N/A*	64	S	bi-functional	(52) (53)
012-12	carrier	N/A*	65	S	bi-functional	(52) (53)
014-24	carrier	N/A*	66	S	bi-functional	(52) (53) (25, 27)
016-24	carrier	N/A*	65	S	bi-functional	(52) (53)
017-02	carrier	N/A*	61	S	bi-functional	(52) (53)
028-12	carrier	N/A*	68	S	bi-functional	(52) (53)
039-03	carrier	N/A*	63	S	bi-functional	(52) (53)
049-12	carrier	N/A*	69	S	bi-functional	(52) (53)
004-12	carrier	N/A*	63	S	bi-functional	(52) (53)
030-24	carrier	N/A*	63	S	bi-functional	This publication
Y92-1009	unspecified	N/A*	52	S	bi-functioal	This publication
N. polysaccharea						
ATCC43768	carrier	N/A*	29	S	bi-functional	ADBE01000000 (27, 54)
CCUG 18031	carrier	N/A*	29	S	bi-functional	(27)
CCUG 24845	carrier	N/A*	58	S	bi-functional	(27)
CCUG 24846	carrier	N/A*	73	S	bi-functional	(27)
CCUG 27182	unknown	N/A*	75	S	bi-functional	(27)
CCUG 4790	carrier	N/A*	106	S	bi-functional	This publication
N. bergeri						
15883	carrier	N/A*	51	S	bi-functional	(27)
N. cinerea						
ATCC14685	carrier	N/A*	30	F	mono-functional	ACDY02000000 (27, 54)
CCUG 346T	unknown	N/A*	53	F	mono-functional	(27)
CCUG 5746	Human eye	N/A*	54	S	bi-functional	(27)
CCUG 25879	Human urine, kidney malfunction	N/A*	55	F	mono-functional	(27)

CCUG 27178 A	proctitis	N/A*	56	F	mono-functional	(27)	
CCUG 28662	Human eye	N/A*	76	F	mono-functional	(27)	
CCUG 53043 B	Human eye	N/A*	108	F	mono-functional	This publication	
N. subflava							
NJ9703	unknown	N/A*	31	F	mono-functional	ACEO02000000	
CCUG 7826		N/A*	72	F	mono-functional	This publication	
CCUG 24918		N/A*	74	F	mono-functional	This publication	
C102		N/A*	112	F	mono-functional	This publication	
N. mucosa var. heidelbergensis							
CCUG 26878T	carrier	N/A*	57	V	bi-functional	(27)	
CCUG 10421	carrier	N/A*	59	V	bi-functional	This publication	
CCUG 804	carrier	N/A*	70	V	bi-functional	This publication	
F0314	carrier	N/A*	113	V	bi-functional	This publication	
N. weaveri							
CCUG 4007T		N/A*	111	Y	bi-functional	(27)	
N. animalis							
CCUG 808	unknown	N/A*	115	C	bi-functional	This publication	
N. elongata var.							
ATCC29315 (*var. glycolitica*)	carrier	N/A*	32	C	bi-functional	ADBF01000042 (27, 54)	
CCUG 4554 (*var elongata*)	carrier	N/A*	71	V	bi-functional	This publication	
CCUG 30802T (*var. nitroreducens*)	blood	N/A*	109	V	bi-functional	This publication	
CCUG 2043T	carrier	N/A*	110	V	bi-functional	This publication	
N. gonorrhoeae							
DGI2	DGI**	*cnl*	19	S	bi-functional	ACIG00000000	
PID1	PID**	*cnl*	19	S	bi-functional	ABZM0000000	
PID18	PID**	*cnl*	19	S	bi-functional	ABZL00000000	

PID332	PID**	*cnl*	19	S	bi-functional	ABZO00000000
SK-92-679	DGI**	*cnl*	19	S	bi-functional	ABZP00000000
SK-93-1035	DGI**	*cnl*	19	S	bi-functional	ABZQ00000000
FA1090	DGI**	*cnl*	17	S	bi-functional	NC002946
NCCP11945	uncomplicated gonorrhoea	*cnl*	19	S	bi-functional	CP001050 (55)
MS-11	uncomplicated gonorrhoea	*cnl*	19	S	bi-functional	ABZK00000000
1291	urethral swab	*cnl*	19	S	bi-functional	ABZF00000000
35/02	uncomplicated gonorrhoea	*cnl*	82	S	bi-functional	ABZG00000000
FA19	uncomplicated gonorrhoea	*cnl*	19	S	bi-functional	ABZJ00000000
F62	uncomplicated gonorrhoea	*cnl*	19	S	bi-functional	ADAA00000000
PID24	PID**	*cnl*	19	S	bi-functional	ABZN00000000
DGI18	DGI**	*cnl*	19	S	bi-functional	ABZH00000000
FA6140	unknown	*cnl*	19	S	bi-functional	ABZI00000000

*ND = not detected either experimentally by serogrouping antibody or by whole genome sequencing as the contigs for this region did not assemble an array of sufficient length to include the flanking regions. N/A = not applicable, serogrouping is not performed for neisserial species other than *N. meningitidis*.

**DGI: Disseminated gonococcal infection; PID: pelvic inflammatory disease
"*Neisseria gonorrhoeae* group Sequencing Project, Broad Institute of Harvard and MIT (http://www.broadinstitute.org/)"
DGI: Disseminated gonococcal infection; PID: pelvic inflammatory disease
"*Neisseria gonorrhoeae* group Sequencing Project, Broad Institute of Harvard and MIT (http://www.broadinstitute.org/)"

Supplementary Table S2. Association of GalE1 functionality with Serogroup

Serogroup	Mono-functional GalE1	Bi-functional GalE1	**Total**	***p*-value***
A	16	1	**17**	0.3791
B	678	21	**699**	<0.0001
C	41	0	**41**	0.0591
E	0	31	**31**	<0.0001
W	119	1	**120**	0.0017
Y	230	22	**252**	0.0326
**Total**	**1084**	**76**	**1160**	-

*The *p*-values were calculated using the Fisher's exact test

Supplementary Table S3. Alleles of *cps* locus (A) and MLST alleles (B) used in the calculation for recombination and mutation rates using ClonalFrame.

Table A: Cps alleles

	Allele integer (prevalence in clonal complex)*											
Clonal Complex	NEISS044	GalE1	CtrA allele	CtrB allele	CtrC allele	CtrD	CtrG allele	GalE2	CtrE allele	CtrF allele	NEIS0069	TEX
ST-1	2(94.1%)	39(88.2%)	20(88.2%)	8(82.4%)	2(82.4%)	2(88.2%)	No Value(100%)	30(88.2%)	34(35.3%)	6(76.5%)	3(35.3%)	3(94.1%)
ST-11(C)	1(97.2%	27(20.1%)	5(19.6%)	3(17.3%)	1(22.9%)	6(92.7%)	1(17.9)	8(97.8%)	45(72.6%)	4(74.3%)	1(21.8%)	52(20.1%)
ST-162	70(83.3%)	43(91.7%)	5(83.3%)	3(83.3%)	7(87.5%)	2(100%)	20(91.7%)	7(87.5%)	27(91.7%)	4(100%)	9(83.3%)	143(62.5%)
ST-174	91(100%)	131(91.7%)	2(91.7%)	54(91.7%)	1(100%)	2(95.8%)	20(91.7%)	23(100%)	136(83.3%)	107(83.3%)	64(79.2%)	154(95.8%)
ST-22	167(66.7%)	8(80.0%)	5(53.3%)	3(33.3%)	1(33.3%)	89(33.3%)	20(86.7%)	13(60.0%)	5(60%)	5(100%)	4(93.3%)	205(33.3%)
ST-23	92(49.8%)	38(98.3%)	2(97%)	2(97.9%)	3(96.6%)	5(87.6%)	20(99.1%)	6(90.1%	45(89.3%)	4(90.1%)	9(93.6%)	14(87.6)
ST-269	31(83.8%)	120(79.2%)	2(65.4%)	2(72.7%)	3(62.7%)	11(61.5%)	13(75.0%)	7(88.1%)	135(58.5%)	106(58.5%)	5(61.2%)	147(54.6%)
ST-32(B)	3(100%)	2(88.0%)	93(20.0%)	61(20.0%)	67(20.0%)	1(68.0%)	6(68.0%)	26(84.0%)	21(84.0%)	2(92%)	4(84.0%)	4(76.0%)
ST-41/44	11(80.4%)	21(78.5%)	2(69.1%)	2(73.8%)	3(72.3%)	12(66.0%)	5(91.6%)	5(94.2%)	43(86.1%)	4(90.3%)	10(85.3%)	26(55.2%)
ST-60 (E)	32(77.8%)	14(68.9%)	6(68.9%)	4(68.9%)	6(68.9%)	8(69.9%)	No Value(68.9%)	8(95.6%)	9(88.9%)	13(73.3%)	10(91.1%)	9(77.8%)

*Prevalence of each allele in each clonal complex was determined using the PubMLST database. The values depicts the percentages obtained at the time of writing.

Table B: MLST alleles

**Clonal Complex**	*abcZ*	*adk*	*aroE*	*fumC*	*gdh*	*pdhC*	*pgm*
ST-1	1(100%)	3(100%)	1 (94.1%)	3(70.6%)	1(100%)	1 (94.1%)	3(94.1%)
ST-11	2(95.5%)	3(100%)	4(100%)	3(99.4%)	8(98.9%)	4(100%)	6(99.4%)
ST-162	1(95.8%)	5(95.8%)	13(95.8)	53(95.8%)	26(91.7%)	41 (95.8%)	3(91.7%)
ST-174	6(100%)	5(100%)	173(100%)	13(91.7)	5(100%)	24(100%)	17(100%)
ST-22	11(66.7%)	5(100%)	18(93.3%)	17(66.7%)	11(100%)	24(93.3%)	21(100%)
ST-23	12(72.5%)	5(98.2%)	18(97.9%)	9 (97.4%)	11(98.7%)	9(98.7%)	17(99.1%)
ST-269	4(99.2%)	10(98.8%)	15 (34.6%)	5(61.9%)	38(53.1%)	11(98.5%)	9(92.7%)
ST-32	4(92%)	10(96%)	5(96%)	4(80%)	6(96%)	3(96%)	8(96%)
ST-41/44	3(82.5%)	6(88.5%)	9(71.2%)	5(81.7%)	9(60.5%)	6(91.1%)	9(85.9%)
ST-60	17(95.6%)	5(86.7%)	19(82.2%)	17(80%)	3(86.7%)	26(88.9%)	2(91.1%)

Supplementary Table S4. Summary of capsule switching events in 90 meningococcal isolates.

Original profile<br>Cc/ ST/ [*galE1* allele-(serogroup)-*galE2* allele] (number of isolates)	Serogroup switched profile<br>cc/ ST / [*galE1* allele-(Serogroup conversion)-*galE2* allele] (number of isolates)
cc 11/ ST 11 / [10-(W)-8] (n=103)	cc 11/ST 11 – [43-B-8] (n=1)
	cc 11 /ST 11 – [127-B-8] (n=1)
	cc 11/ST 11 –[120-B-8] (n=2)
	cc 11/ST 11 –[42-B-8] (n=1)
	cc 11/ ST 11 – [42-C-8] (n=1)
	cc11/ ST11 - [27-C-5] (n=2)
	cc11/ ST11 - [27-C-8] (n=58)
	cc 11/ ST11 – [1-B-1] (n=1), /- [1-C-1] (n=2)
Cc 60/ ST60 [14-(E)-8] (n=6)	cc60/ ST60 – [119-B-8] (n=1)
	cc 60/ST60 – [211-B-8] (n=1)
	cc 60/ST 60 – [43-B-7] (n=1)
	cc 60/ST60 – [127-B-8] (n=1)
	cc 60/ST60 – [153-B-8] (n=3)
	cc60/ST60 – [27-B-5] (n=1)
	cc60/ ST60 –[153-B-8] (n=1)
	cc60/ST60 – [120-B-8] (n=1)
	cc60/ ST60 – [23-B-8] (n=1)
	cc60/ ST60 – [277-B-8] (n=1)
	cc60/ ST5103 – [127-B-8] (n=1)
	cc60/ ST7788 – [43-B-8] (n=1)
	cc60/ ST9818 – [21-B-8] (n=1)
	cc60/ST9821 – [142-B-8] (n=1)
Cc 174/ST1466 [131-75(Y)-23] (n=21)	cc174/ ST1466 – [27-C-23] (n=2)

Supplementary Table S5. Strains used in this study.

**Strain Name**	**Genotype**	**Ref**
Neisseria meningitidis strain *MC58*	*B:15:P1.7,16:L3	[55]
Neisseria gonorrhoeae strain *FA1090*		[56]
Escherichia coli BL21-DE3 Rosetta	F^- *ompT hsdS*$_B$(R_B^- m_B^-) *gal dcm* λ(DE3 [*lacI lacUV5*-T7 gene 1 *ind1 sam7 nin5*]) pLysSRARE (Cm^R)	Novagen
Escherichia coli DH5α	*fhuA2 lac(del)U169 phoA glnV44 Φ80' lacZ(del)M15 gyrA96 recA1 relA1 endA1 thi-1 hsdR17*	[57]
CKEC729	DH5α expressing gonococcal GalE_17::Hisx6 from pET15b	This study
CKEC730	DH5α expressing meningococcal GalE_2::Hisx6 from pET15b	This study
CKEC733	DH5α expressing GalE_2(F300S)::Hisx6 from pET15b	This study
CKEC734	DH5α expressing GalE_17(S299F)::Hisx6 from pET15b	This study
CKEC771	DH5α expressing GalE_236::Hisx6 from pCMK771	This study

* Nomenclature is derived from serological typing scheme for capsule polysaccharide (serogroup B):porin B variant (2B or 15):porin A variant (P1.2,5 or P1.1,16) :lipooligosaccharide immunotype (L2 or L3).
